# Predictors of Atrial Fibrillation Recurrence after Catheter Ablation: Data from the German Ablation Registry

**DOI:** 10.1038/s41598-017-16938-6

**Published:** 2017-11-30

**Authors:** A. Sultan, J. Lüker, D. Andresen, K. H. Kuck, E. Hoffmann, J. Brachmann, M. Hochadel, S. Willems, L. Eckardt, T. Lewalter, J. Senges, D. Steven

**Affiliations:** 1 University Heart Center Cologne, Dept. of Electrophysiology, Cologne, Germany; 2 Elisabeth Hosp. Berlin, Berlin, Germany; 3 Asklepios Hosp. St. Georg Hamburg, Hamburg, Germany; 4 Hosp. Munich, Munich, Germany; 5 Hosp. Coburg, Coburg, Germany; 6 IHF Ludwigshafen, Ludwigshafen, Germany; 7 University Heart Center Hamburg, Dept. of Electrophysiology, Hamburg, Germany; 8 University Hosp. Münster, Dept. of Electrophysiology, Münster, Germany; 9 Dept. of Medicine-Cardiology, P. Osypka Heart Center Munich, Munich, Germany

## Abstract

Catheter ablation (CA) for atrial fibrillation (AF) has emerged as a widespread first or second line treatment option. However, up to 45% of patients (pts) show recurrence of AF within 12 month after CA. We present prospective multicenter registry data comparing characteristics of pts with and without recurrence of AF within the first year after CA. This study comprises all pts with complete follow-up one year after CA (1-y-FU; n = 3679). During 1y-FU in 1687 (45.9%) pts recurrence of AF occurred. The multivariate analysis revealed female sex and AF type prior to the procedure as predictors for AF recurrence. Furthermore, comorbidities such as valvular heart disease and renal failure as well as an early AF relapse were also predictors of AF recurrence during 1-y-FU. However, despite an AF recurrence rate of 45.9%, the majority of these pts (72.4%) reported a significant alleviation of clinical symptoms. In conclusion in pts with initially successful CA for AF female sex, AF type, in-hospital AF relapse and comorbidities such as renal failure and valvular heart disease are independent predictors for AF recurrence during 1-y-FU. However, the majority of pts deemed their interventions as successful with significant reduction of symptoms irrespective of AF.

## Introduction

Catheter ablation (CA) of atrial fibrillation (AF) has become a safe and well-established treatment option in patients with symptomatic AF^1,2^. High acute success rates are achievable but durable efficacy of previously successful CA for AF still remains a major challenge. Therefore, success rates vary between 60% to 80%, for paroxysmal AF (PAF), depending on ablation strategies and between 50% to 60% for persistent AF (persAF)^3–6^.

Pertaining to possible recurrence rates of up to 50% in persAF patients as shown in the STAR AF II trial, patient selection is crucial for an optimal CA outcome^6^. Within the last decade several studies identified factors such as AF duration, age and AF cycle length, but also structural heart disease as predictors for arrhythmia recurrences after an initially successful CA^7–11^. Given the moderate above mentioned success rates, it would facilitate patients‘ selection for CA with a favorable outcome, if characteristics predicting arrhythmia recurrences would be available. Although all listed studies were well conducted and therefore identified predictors are reliable, collected data reflect mainly single center data imposed by very well experienced operators. Also, patients undergoing AF ablation in these centers are potentially very well selected due to the expertise of the operating and out-clinic team. Thus, the aim of our study was to identify more general, preferably not selective patients groups based on data entry of the German nation-wide ablation registry as well as including moderate volume centers potentially presenting a more “real-life” oriented view on realistic outcome data after CA for AF. We furthermore evaluated the question, whether despite of a formal AF recurrence (documented AF duration >30 seconds) patients still experienced a benefit from the ablation procedure, e.g. reporting a symptom reduction, capacity improvement or less weighted AF episodes.

## Methods

### Participating centers

The German ablation registry incorporated ablation data from 3703 patients undergoing CA for AF in different hospitals in Germany. The expertise of ablation centers varied from smaller centers (<300 CA/y) up to high volume centers (>1000 CA/y). A total of 40 German electrophysiological departments contributed data on AF ablations into this prospective multicenter registry. All patients included in this study gave written informed consent for their participation and follow-up contacts after ablation. The ethics committee of the Landesaerztekammer Rheinland-Pfalz approved the study in 2007 and all methods were performed in accordance with the relevant guidelines and regulations.

### Registry data management

Project development and management as well as data collection were conducted by the “Institut für Herzinfarktforschung (IHF)” (Ludwigshafen, Germany). Data acquisition and documentation was carried out using an online case report form as published previously^12,13^. All obtained information was confidential and transmitted in an encoded secure socket layer mode.

### Study population

This registry consists of data collection for CA for symptomatic AF between 2008 and 2014. All patients in this cohort showed symptomatic AF despite an optimal antiarrhythmic drug (AAD) regime. Patients who had died previous to the 1-year follow-up were excluded from the present analysis. The resulting cohort of 3679 follow-up survivors (2461 (66.7%) men; age 60.7 ± 10.3) was analyzed for AF recurrence during 1-y-FU or no AF recurrence during 1-y-FU.

For the overall cohort demographic and clinical information was obtained including age, sex and comorbidities such as hypertension, valvular disease, renal insufficiency, coronary artery disease, stroke and the presence of cardiac devices (PM, ICD, CRT). Furthermore, the IHF obtained patients‘ AF history, type of AF ablation (cryoballoon, radiofrequency ablation, others) and procedural data.

### Electrophysiological Study and Ablation Procedure

The ablation strategy for all patients was in accordance to the ablation centers‘ standards. However, proof of electrical PV entrance block using a circumferential mapping catheter was required for all veins. Further ablation beyond PVI, including ablation of complex fractioned atrial electrograms (CFAEs) in the left and right atrium and/or deployment of linear lesions, if necessary, was left to the executing centers.

Aside from conventional mapping mainly two different 3-D cardiac mapping systems were used: 1. CARTO^TM^ (Biosense Webster, USA) in 64.5% of patients and 2. Ensite Navx^TM^ (St. Jude Medical, USA) in 24.8% of patients. A cryoablation (cryo) was performed in 20.5% of patients.

In a minority (26.3%) of patients additional cardiac imaging such as MRI (4.3%), CT scan (19.6%) or intra-cardiac echocardiography (ICE) (1.7%) was performed.

### Complications

In-hospital complications were categorized into minor, moderate or major acute procedure related complications. Minor was defined as minor bleeding without intervention, newly developed bundle branch or AV block not prolonging the hospital stay.

A transient ischemic attack (TIA), transient resuscitation, femoral vein access related complications (hematoma, fistula, aneurysm) requiring transfusion or surgical correction, AV block III° and/or phrenic nerve injury were defined as moderate non-fatal complications according to previous publications on German registry data^12,14^. Major complications were defined as stroke, pericardial tamponade, myocardial infarction, atrio-esophageal fistula and clinically relevant PV stenosis.

The occurrence of possible MACE (death, myocardial infarction) or MACCE (death, myocardial infarction or stroke) complications and the possible necessity for repeat ablation were evaluated before discharge and during 12 months FU.

### Follow Up

During 1 year follow up (FU) patients were seen in accordance to usual FU intervals of each ablation center. Furthermore, the 1-year follow-up including personal telephone interview, 12 lead ECG and 24 hours Holter ECG was also conducted and largely funded by the IHF, with additional support by unrestricted grants from Medtronic, Biosense Webster, and Biotronik. All patients were evaluated for late complications, AF symptoms and ongoing medication. Patients were asked to categorize their symptoms as either unchanged, worsened or improved. Furthermore, interrogation regarding a potential improvement of quality of life and changes in physical capacity was performed. Repetitive FU evaluations were performed every three-month. Recurrence of AF was defined as a documented AF episode of at least 30 seconds. After occurrence of AF during 1-y-FU a repeat procedure was optional.

### Statistical Analysis

Categorical data are presented as absolute counts and percentages, metrical variables as means with standard deviation. For comparison of patient’s baseline and outcome characteristics between both outcome groups the CHI^2^ or Mann-Whitney-Wilcoxon test was used. The binary variables were compared between groups using the Pearson chi-square test and odds ratios with 95%-confidence intervals were calculated.

Predictors of recurrence of arrhythmia during 1-y FU were analyzed using multiple logistic regressions. In addition to age, the following baseline characteristics that showed a significant association with recurrence in univariate comparisons were included in the model: gender, type of AF, valvular heart disease, renal failure, in-hospital relapse of AF and presentation NYHA class II or higher.

A p-value of less than 0.05 was considered statistically significant. The statistical analysis was performed at the biometrics department of the IHF using SAS software release 9.3 (SAS Institute, Inc., Cary, North Carolina. U.S.A.).

## Results

### Patient Characteristics

A total of 3703 patients undergoing CA for AF in 40 participating German centers were enrolled in the registry. Out of these, 1 patient died in hospital, due to severe heart failure and 23 during FU. Therefore information on arrhythmia recurrence of 3679 (99.3%) patients over a time course of at least 1-year follow-up (with a median of 463 days) was obtained. During 1-y-FU a total of 1687 (45.9%) patients had AF recurrence (Table 1).

**Table 1:** Baseline data of the overall study cohort and with 1-y-FU (divided in patients with and without AF recurrence during 1-y-FU).

Patients’ baseline characteristics					
	All patients	AF recurrence	No AF recurrence	p-value	OR (95%-CI)
**Number of patients**	3679	1687 (45.9%)	1992 (54.1%)		
Male	66.9%	64.0%	69.3%	<0.001	0.79 (0.69–0.90)
Female	33.1%	36.0%	30.7%	<0.001	1.27 (1.11–1.46)
Age (MW)	60.8 ± 10.1	61 ± 9.9	60.4 ± 10.7	0.23	
**Comorbidities**					
Diabetes mellitus	7.9%	8.1%	7.8%	0.80	1.03 (0.81–1.31)
Renal insufficiency*	2.8%	4.4%	1.4%	**0.027**	3.35 (1.08–10.41)
Hypertension*	61.5%	62.4%	60.7%	0.67	1.07 (0.77–1.50)
History of stroke*	4.8%	4.7%	4.8%	0.99	1.00 (0.47–2.13)
CHD	16.9%	15.9%	17.8%	0.13	0.87 (0.74–1.04)
History of myocardial infarction	3.0%	3.3%	2.8%	0.37	1.19 (0.81–1.73)
Valvular disease	8.2%	9.2%	7.3%	**0.035**	1.29 (1.02–1.63)
Cardiomyopathy (CM)	3.4%	3.6%	3.3%	0.69	1.08 (0.75–1.54)
- Dilative CM	73.1%	70.0%	75.8%	0.47	0.75 (0.34–1.64)
- Hypertrophic CM	26.9%	30.0%	24.2%	0.47	1.34 (0.61–2.95)
**LV-Function**					
LV-Fx: severely impaired (<=40%)	5.0%	4.7%	5.3%	0.53	0.84 (0.60–1.18)
NYHA 2**	34.4%	40.0%	29.6%	**0.011**	1.58 (1.36–1.84)
**AF History**					
Paroxysmal (PAF)	65.9%	64.1%	67.4%	**0.034**	0.86 (0.75–0.99)
Persistent (PersAF)	26.6%	26.9%	26.4%	0.75	1.02 (0.88–1.19)
Long standing persistent	7.5%	9.0%	6.2%	**0.001**	1.51 (1.18–1.93)

Showing significant differences in sex, AF duration and comorbidities.

Displayed: Percentage and number of pts or MV with SD; p-value: Chi² test or Mann-Whitney-Wilcoxon test.

CI: Confidence-interval, OR: Odds Ratio.

*Information obtained since October 2008; **Reported for patients with organic heart disease.

Patients with AF recurrence during 1-y-FU were more often females (36.0% vs. 30.7%; p < 0.001) and more likely to have longstanding persistent atrial fibrillation (6.2% vs. 9.0%; p < 0.001). Furthermore, comorbidities such as renal insufficiency and valvular heart disease were significantly more frequent in patients with AF recurrence (renal insufficiency 1.4% vs. 4.4%; p = 0.03; valvular disease 9.2% vs. 7.3%; p = 0.04). Also, patients with AF recurrence were more likely to present in NYHA class II or more than patients without AF recurrence during FU (NYHA II 40% vs. 29.6%; p = 0.011). Patients in SR during 1y- FU were more likely to have paroxysmal AF (67.4% vs. 64.1%; p = 0.03) (Table 1).

#### Ablation procedure

In the overall cohort no difference was found regarding number of procedures. The average procedure number was 1.2 procedures including the index and potential repeat-procedures during FU. The vast majority of patients underwent a single procedure. For 801 (47.5%) out of 1687 patients with AF recurrence a second procedure was performed.

Radiofrequency ablation (RF) was used predominantly (77.2% vs. 78.2%; p = 0.49). A cryoablation (cryo) was performed in 20.5% of patients.

No difference regarding acute procedural success, defined as PV isolation, was detectable in patients with and without AF recurrence (96.6% vs. 96.4%; p = 0.70) regardless if RF or cryo was used. Also, the procedure duration was similar (182.7 ± 71.0 vs. 182.5 ± 70.2 min; p = 0.89) irrespective of the energy source. The maximum delivered energy in patients with AF recurrence was significantly lower as opposed to patients without AF recurrence (35 W vs. 37 W; p < 0.01) along with the RF application duration, which was shorter in patients with AF recurrence during 1-y-FU (1320–3279 sec. vs. 1500–3540 sec., p < 0.01).

Patients with AF recurrence showed significantly higher fluoroscopy dosages than patients without AF recurrence 3990 (2101–7900) vs. 3540 (1803–6699) (cGy)*cm^2^; p < 0.001). The fluoroscopy time was equal in both groups (28 (20–42) vs. 28 (18–45) min; p = 0.56).

Patients with AF recurrence during 1-y-FU were more likely to have a history of in-hospital AF-relapse after successful CA (11.1% vs. 5.8%, p < 0.001).

#### Procedural safety

The incidence of acute MACE (0.1% vs. 0.1%; p = 1.00) or MACCE (0.3% vs. 0.3%; p = 1.0) was similar in patients with and without AF recurrence during 1-y-FU, furthermore no difference in days of hospital stay was detectable (4d(3;6) vs. 4d(3;5); p = 0.87). Also, no difference was noted in the overall complication rate in patients with and without AF recurrence (2.5% vs. 2.7%; p = 0.54) independent of the energy source used (RF or cryo).

#### Antiarrhythmic drug medication and oral anticoagulation

Significantly more patients with AF recurrence during 1-y-FU were on antiarrhythmic drugs (AAD) by the time of discharge as compared to patients without AF recurrence (59.0% vs. 53.6%; p < 0.001). Regarding oral anticoagulation no differences were detectable in patients with or without AF relapse during 1-y-FU.

### Follow Up

Patients with AF recurrence were more frequently on AAD as compared to patients free from AF (43.2% vs. 24.2%; p < 0.001) after 1-y-FU.

No differences were detectable in the occurrence of MACE (death, myocardial infarction) or MACCE (death, MI, stroke) during 1-y-FU (0.2% vs. 0.1%; p = 0.24). Minor non-fatal complications such as syncope and minor bleeding were more frequent in patients with AF recurrence during 1-y-FU (10.2% vs. 6.6%; p < 0.001). Rates of ablation-associated “late-complications” such as PV stenosis, phrenicus lesions or atrio-oesophageal fistula were similar for patients with or without recurrence of AF (0.9% vs. 0.5%; p = 0.14). Only one atrio-oesophageal fistula occurred in the total cohort. During FU significantly more patients with AF recurrence received a pacemaker mainly due to worsening of preexisting sick-sinus-syndrome or symptomatic brady-arrhythmia (4.2% vs. 1.3%; p < 0.001). Significantly more patients with AF recurrence during 1-y-FU were scheduled to undergo a second ablation procedure (46% vs. 2.6%; p < 0.001).

### Multivariate analysis

The multivariate analysis revealed female sex (OR 1.27; CI 1.11–1.46; p < 0.001) and AF type (long. pers. AF vs. PAF; OR 1.51; CI 1.18–1.93; p = 0.001) as strong predictors for AF recurrence during 1 y FU. Furthermore, comorbidities such as valvular heart disease and renal failure were also predictors of AF recurrence during FU (OR 1.29; CI 1.02–1.63; p = 0.04; OR 3.35; CI 1.08–10.41; p = 0.03) (Table 2).

**Table 2:** Multivariate analysis of patients baseline data for pts with and without AF recurrence during 1-y-FU.

	Odds ratio	95% CI	p-value
Age [per 10-year increase]	1.02	0.96–1.09	0.53
Female vs. Male	1.27	1.11–1.46	<0.001
PersAF vs. PAF	1.02	0.88–1.19	0.29
Long standing pers. AF vs. PAF	1.51	1.18–1.93	0.001
Valvular heart disease	1.29	1.02–1.63	0.04
Renal failure	3.35	1.08–10.41	0.03
NYHA II+ in organic heart disease	1.58	1.36–1.84	0.001
In-Hospital AF recurrence	2.03	1.60–2.59	0.001

Revealing female sex, in-hospital AF relapse, AF duration and comorbidities as predictors of AF recurrence during 1-y-FU.

Occurrence of an already in-hospital AF relapse after successful CA was also a predictor of AF recurrence during 1-FU (OR 2.03; CI 1.60–2.59; p < 0.001).

### Rehospitalization

Rehospitalization during 1-y-FU due to worsening of preexisting cardiovascular diseases or any other cause was more frequent in patients with AF recurrence during 1-y-FU compared to patients without AF recurrence (69.1% vs. 24.8%; p < 0.001). Also, the time to rehospitalization after discharge was significantly shorter in patients with AF recurrence (155d; 73–289d vs. 202d; 89–334d; p < 0.001).

### “Repeat procedure within FU”

During 1-y-FU in 801 (47.5%) out of 1687 patients with AF recurrence a repeat ablation procedure was performed. The remaining 886 (52.5%) patients refused a second procedure despite AF recurrence. No differences were detectable regarding age, gender or comorbidities. Patients undergoing a repeat procedure during FU more often suffered from long standing persistent AF than from paroxysmal AF compared to patients not undergoing a repeat procedure (10.1% vs. 6.1%; OR 1.19–2.04; p < 0.001 for long standing AF and 62.4% vs. 67.1%; OR 0.69–0.96; p < 0.013 for paroxysmal AF). The occurrence of complications was equal to the index procedure.

Patients with a repeat ablation during FU were more often on AADs (41% vs. 30.9%; p < 0.001). Also, patients undergoing a repeat procedure showed a very low recurrence rate of AF as compared to patients not undergoing a repeat procedure (AF recurrence: 6.2% vs. 68%; p < 0.001). AF type (long standing persistent AF) seems to be the only predictor for a repeat procedure (OR: 1.56 (1.19–2.04), p < 0.001).

## Discussion

### Main finding

This analysis of the German ablation registry of 3703 patients after initially successful CA for AF, aimed to reveal predictors of AF recurrence during 1y-FU and to identify differences between those patients with and without AF recurrence. As a main finding the multivariate analysis of baseline data identified female sex and AF type as independent predictors of AF recurrence. Furthermore, comorbidities such as impaired cardiac and renal function were also classified as predictors for AF recurrence in these patients, emphasizing the importance of these conditions when planning CA for AF. Notably, an in-hospital relapse of AF after successful CA was also a predictor for AF recurrence during 1-y-FU.

These real world data show a success rate of 54.1% after one procedure during 1-y-FU for AF. However, the vast majority (72.4%) of patients with AF relapse during 1-y-FU reported a significant reduction of symptoms and therefore improvement of quality of life although no long-term CA success was achieved. In less then half (47.5%) of patients with AF recurrence a repeat ablation was performed.

### Procedural findings

Although no difference was detectable in acute procedural success rates, significantly longer RF application time and higher maximum energy levels were noted in patients without AF recurrence during 1-y-FU indicating a link between energy delivery and outcome^4^. Furthermore, the fluoroscopy time was lower in patients without AF recurrence during 1-y-FU. One can speculate that this relates to less complex anatomical circumstances leading to better outcomes.

The usage of cryo for PVI in the overall cohort and its ablation success rates are in accordance with recently published data from the Fire and Ice Trial^15^.

### Efficacy and Safety

The rate of incomplete PVI during the index procedure of 1.5% in this registry may be due to the fact that not only experienced high volume centers but also moderate volume centers contributed their data to this nationwide registry. However, these success rate findings despite a primary complete PVI (98.5% vs. 97.8%) are well in line with previous studies and meta-analysis^16^. Furthermore, the procedure related complication rate in the registry cohort ranging from 3.4% for moderate to 1.2% for severe non-lethal complications and 0.1% for MACCE (equally in both groups) is below published data from a worldwide survey and a meta-analysis reporting a complication rate of 4.5% up to 4.9%^16,17^. A more recent study showed an overall complication rate of 6.29% in patients undergoing CA for AF. The study revealed a strong association between operator and hospital volume and the occurrence of adverse events, emphasizing the importance of experienced centers and operators^18,19^.

### Outcome and Follow up

Due to the heterogeneity of AF types included in the registry (up to 40% pts with persistent AF) and not equally experienced centers an overall single procedure AF free survival of 54.1% during 1-y-FU is in range with previous studies reporting success rates from 50% for persistent and long standing persistent AF patients and up to 80% for paroxysmal AF depending on the ablation strategy^4,5,7,20^. Individual ablation strategies, different energy sources and catheter technologies influence CA outcome^4,21^. Therefore, the variety of different CA settings of the participating centers has possibly contributed to these rather moderate success rates, however they may more realistically display real world data.

As seen in previous single center studies, the analysis of the German ablation registry also revealed female sex as well as long standing persistent AF as independent predictors for AF recurrence after an initially successful CA^3,10,22^. Female sex has been described as and an independent risk factor for recurrence of AF after CA of AF^10,23,24^, in accordance a recent MRI study revealed that female sex is associated with a higher burden of atrial fibrosis in patients with AF and therefore increasing AF recurrence^25^.

Due to the complexity of AF classification long standing persistent AF patients in this cohort reflect a group of patients with a significant longer AF duration as opposed to patients with possibly early electrical CVs and therefore classified as persAF patients. Possibly, AF duration is more valuable and accurate than the standard AF classification. Hence, a significant difference was seen between patients with PAF and long standing persistent AF.

Although AF is more prevalent in older patients and in patients with CHD the registry data did not reveal these as independent predictors for AF recurrence, these findings are in line with earlier studies^9,24^. In only some previous studies an age >60 y was revealed as a risk factor of AF recurrence^23^. However, the mean age in this cohort was 60.7 ± 10.3 y and therefore a possibly rather young cohort. More interestingly, comorbidities such as heart failure and impaired renal function were identified as predictors for AF recurrence in the registry cohort. These findings do correspond with previous single center data emphasizing the importance of comorbidities in the setting of AF^3,26^. They furthermore support the use of earlier identified non-invasive risk stratification tools (i.e. AF duration, cardiac and renal function) to identify eligible patients for CA^9^. Therefore, a more effective patient selection should be achievable by application of factors predicting AF recurrence as evaluated in the present study, especially in patients suffering from long standing persistent AF.

As recently shown in the ADVICE trial these large cohort data also highlight the importance of early AF relapses (ER) during the blanking period and their possible prognostic value for long-term success rates after CA. In this cohort more patients with AF recurrence during 1-y-FU already experienced an ER during the hospital stay (11.1% vs. 5.8%; p < 0.001)^27^. Therefore, current blanking period recommendations are potentially debatable^27^.

It was noteworthy in this registry data that although 45.9% of patients experienced AF recurrence during FU, the majority of these patients (72.4%) deemed their ablation as successful mostly due to an AF burden reduction and therefore possibly improved comorbidities and quality of life^28^. This finding also reflects our real life setting showing that although single procedure outcomes are still improvable an AF burden reduction per se also may be sufficient for some patients and therefore CA should be considered.

Regarding ablation strategies a constant improvement for paroxysmal AF CA is notable, however the best ablation strategy for persistent and long standing persistent AF remains to be determined and was therefore heterogeneously used in this large patient cohort^4,6,15^.

## Limitations

The presented data is obtained from a nationwide registry of 40 participating centers; therefore the data quality is heterogeneous due to possible local differences in ablation strategies, patient care and data collection as well as voluntary data entry. No formal test hypotheses have been specified a priori and no control group of patients without catheter ablation is available. Therefore, the results should be interpreted in a descriptive, not in a confirmatory sense.

However, registry data are of tremendous importance since these data reflect the daily real world experience in all participating ablation centers and therefore should be investigated and evaluated to obtain insights from less experienced ablation sites and to possibly establish universally valid recommendations.

### Potential clinical implications

Given the registry data and data from past studies, patient selection is crucial to obtain low recurrence rates. Despite improvement of ablation tools and strategies a consistent CA approach might be of potential value for long-term success. As revealed in the registry, a non-invasive risk stratification with focus on comorbidities such as renal failure and valvular heart disease especially in patients with longer AF duration might help to distinguish eligible from non-eligible patients to undergo CA^9^. Furthermore, the decision on a repeat procedure should also depend on patients’ subjective outcome rather than AF recurrence alone.”

## Conclusion

The German ablation registry data showed that AF type, female gender and in-hospital AF relapse are strong predictors for AF recurrence as well as comorbidities such as impaired cardiac and renal function. Despite the moderate CA success rates in completely abolishing AF, the majority of these patients still experience a significant symptom reduction after CA due to AF burden reduction.
